# Examining the Rate of Clostridioides (Formerly Clostridium) Difficile Infection Pre- and Post-COVID-19 Pandemic: An Institutional Review

**DOI:** 10.7759/cureus.20397

**Published:** 2021-12-13

**Authors:** Sujani Yadlapati, Simone A Jarrett, Kevin B Lo, Jamie Sweet, Thomas A Judge

**Affiliations:** 1 Gastroenterology and Hepatology, Cooper University Hospital, Camden, USA; 2 Internal Medicine, Einstein Medical Center Philadelphia, Philadelphia, USA; 3 Infectious Disease, Einstein Medical Center Philadelphia, Philadelphia, USA; 4 Gastroenterology, Cooper University Hospital, Camden, USA

**Keywords:** c. diff, nosocomial infections, hospital-acquired infection, personal protective equipment (ppe), covid-19 outbreak

## Abstract

Background/ Rationale

*Clostridioides difficile* infection (CDI) is transmitted via the fecal-oral route and is implicated in antibiotic-associated colitis. Similar to CDI, patients with coronavirus disease 2019 (COVID-19) require early identification and isolation, appropriate personal protective equipment, and environmental disinfection to prevent further transmission. In light of this similarity between isolation and protective requirements to prevent transmission of these diseases, we aim to investigate whether there was a decrease in the incidence of CDI during the peak periods of the COVID-19 pandemic compared to historical rates.

Methods

This is a single-center retrospective analysis of the rates of CDI in our institution. COVID-19 time periods were identified from March 2020 to January 2021 and peak periods (with >50 active patients per day) were defined. The non-COVID-19 periods were July 2017 to February 2020. Rates of CDI were also directly compared across the yearly time period. CDI rates were presented in a per 1000 patient days format. Rates were analyzed per year and during the COVID-19 peaks at our institution. Mann-Whitney U test was used to compare rates between two time periods, while differences across multiple time intervals were analyzed using the Kruskal-Wallis test.

Results

The median (interquartile range [IQR]) of CDI rates of infection per 1000 patient days for the non-COVID time period from July 2017 to February 2020 was 0.34 (0.23-0.45) while COVID time periods had higher 0.44 (0.25-0.51) rates of CDI although this was not statistically significant (p=0.224). However, there was a statistically significant difference (p=0.036) with COVID peak periods having higher rates of CDI 0.49(0.39-0.74) vs 0.34(0.23-0.44). Overall, there was no statistically significant difference in the rates of CDI across years or time periods (p=0.396).

Discussion/Conclusion

There was no difference in the rates of hospital-acquired CDI between COVID-19 and non-COVID-19 time periods at our institution.

## Introduction

Globally, there have been 237 million cases of coronavirus disease 2019 (COVID-19) infection and over 4 million deaths [1]. COVID-19 infection, caused by severe acute respiratory syndrome coronavirus (SARS-CoV-2) commonly causes pulmonary symptoms. However, sufficient clinical evidence now exists to confirm that COVID-19 infection can result in multi-systemic involvement and can often present with extra-pulmonary symptoms. This includes gastrointestinal symptoms such as diarrhea which may be a result of underlying enteritis or colitis. Although a significant population remains asymptomatic, continued transmission from asymptomatic carriers to vulnerable population has been identified [2]. Prior to this pandemic, the most common organism causing hospital-acquired infections across the United States was *Clostridioides difficile* (C. diff) [3]. *C. difficile* infection (CDI) impacts 13 in every 1000 patients and about 75% of cases are considered hospital-acquired [4]. Higher rates of hospital admissions and antibiotic use have been linked with increased rates of healthcare-associated CDI (HA-CDI). Nosocomial CDI is linked to increased morbidity and mortality.

With the emergence of COVID-19, the majority of the hospitals in developed countries such as the United States have seen a change in system operations. Some of these changes include strict infection control measures along with increased use of personal protective equipment (PPE), environmental disinfection, cessation of elective procedures, and reduction in non-COVID-19-related hospital admissions [5]. All these measures indirectly serve to reduce the incidence of nosocomial CDI. On the other hand, patients with confirmed COVID-19 risk exposure to antibiotics and alterations in microbiome, which inadvertently increases risk of CDI.

Patients with COVID-19 require early identification and isolation, appropriate PPE, and environmental disinfection to prevent further transmission. These measures are also required for prevention of nosocomial CDI. Considering this similarity between isolation and protective requirements to prevent transmission of these diseases, we aim to investigate whether there was a decrease in the incidence of CDI during the peak periods of the COVID-19 pandemic compared to historical rates.

This study was previously presented as a poster at the American College of Gastroenterology (ACG) annual meeting held in October 2021.

## Materials and methods

Study design

This was a retrospective study performed at a single tertiary care institution. This study was approved by our institutional review board. Our study examined rates of CDI from July 2017 to January 2021. COVID-19 period was identified as of March 2020 to January 2021 and peak periods (with >50 active patients per day) were defined. The non-COVID-19 time periods were July 2017 to February 2020. Information of positive-C. diff status was obtained from our institutional nosocomial infection database and chart review. This information was utilized to determine C. diff cases per 1000 patient days. Diagnosis of COVID-19 was made using nasopharyngeal swab polymerase chain reaction (PCR). CDI testing was performed via glutamate dehydrogenase (GDH) and enzyme-linked immunosorbent assay (ELISA) for C. diff toxin. CDI was confirmed if both tests were positive. For patients with discordant GDH/ELISA results, PCR was performed as a confirmatory test. Nosocomial CDI in our study was defined as any patient who tested positive for CDI on or after day 3 of hospitalization [3,4].

Inclusion criteria

All adults of age 18 and older admitted to our institution between July 2017 and January 2021 were included in our study.

Exclusion criteria

Patients who were discharged from the emergency department were not included in our study. Those patients who had diarrhea prior to hospital admission or those without a clear diagnosis of nosocomial CDI (failing to fulfill the criteria mentioned above) were excluded. Special populations including non-adults (infants, children, teenagers), pregnant women, and prisoners were not included in our study. The focus of this study was primarily on nosocomial CDI rather than chronic colonization or those patients presenting for recurrent CDI.

Data collection 

The number of inpatients with CDI between July 2017 and January 2021 were identified from our institutional hospital-acquired infection database. We compared the incidence of disease before and after the onset of the pandemic. Collected data was stored appropriately in password-protected database files. Patient identifiers were removed and each patient was assigned an arbitrary study ID number.

Statistical analysis

No sample size determination was made. The sampling method was based on convenience sampling and consisted of all patients with a confirmed diagnosis of C diff in the above described time periods. Rates of CDI were also directly compared across the years. CDI rates were presented in a per 1000 patient days format. Rates were analyzed per year and during the COVID-19 peaks at our institution. Mann-Whitney U test was used to determine rates between two periods while analyzing differences across multiple time periods using the Kruskal-Wallis test.

## Results

Data points observed in our study include CDI rates pre-pandemic and presence of COVID-19 infection and CDI during the pandemic. The COVID-19 time period in our study was defined as March 2020 to January 2021 and the pre-pandemic time period was defined as July 2017 to February 2020. Information on CDI infection rate per month was obtained from our institutional nosocomial infection database. This data was used to determine CDI rates per 1000 patient days. The annual median IQR across the study time period was similar (Table 1). Overall, CDI rate per 1000 patient days pre-pandemic period was 0.34 (0.23-0.45) while the median CDI rate during the COVID period was 0.44 (0.25-0.51). This was not statistically significant, p=0.224 (Table 2).

**Table 1:** Clostridiodes difficile infection rates per 1000 patient days from 2017 to 2021 CDI: *Clostridioides difficile* infection; IQR: interquartile range

	Yearly CDI rates per 1000 patient days			
IQR	2017	2018	2019	2020-2021
25^th^ centile	0.25	0.22	0.14	0.25
75^th^ centile	0.5	0.43	0.50	0.45
Median IQR	0.4	0.32	0.34	0.38

**Table 2:** Clostridioides difficile infection rates per 1000 patient days pre- and post-pandemic IQR: interquartile range

IQR	Pre-pandemic (rates per 1000 patient days)	Pandemic (rates per 1000 patient days)	Peak pandemic period
25^th^ percentile	0.23	0.25	0.39
75^th^ percentile	0.45	0.51	0.74
Median IQR	0.34	0.44	0.49
P value		0.224	0.036

**Figure 1:**
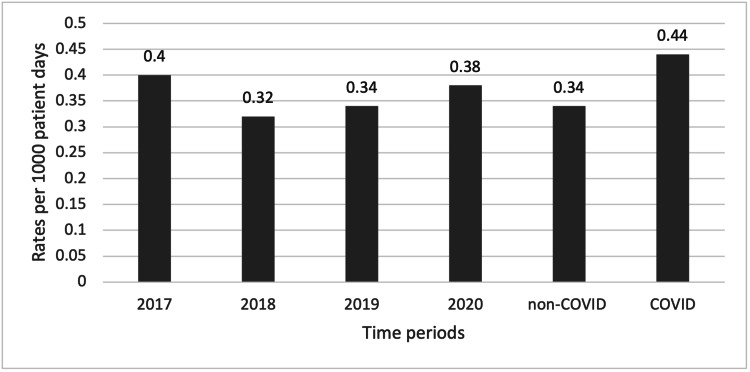
Median Clostridioides difficile infection rates across study time periods COVID: coronavirus disease

Rate of CDI at the peak period of COVID-19 was 0.49 (0.39-0.74) per 1000 patient days. The rate of CDI at the COVID-19 peak period was significantly higher compared to the rate before the pandemic (0.49 vs. 0.34 per 1000 patient days, p=0.036). Overall, there was no significant difference in the rates of CDI across years or time periods p=0.396 (Figure 1).

## Discussion

Based on our data, COVID-19 infection did not have a significant impact on overall CDI rates. However, an increased rate of CDI was observed during peak periods (>50 active COVID-19 patient admissions) of the pandemic. Several other studies published in the recent year have arrived at similar conclusions overall. In a study published by Allegretti et al., CDI rate during the pandemic was lower than the pre-pandemic CDI prevalence of 10% [6]. These findings may emphasize the unintended benefit of reducing nosocomial transmission via aggressive hand washing and donning and doffing protocols [7,8]. In addition, diarrhea is a well-described symptom of COVID-19 infection. Those experiencing diarrhea while admitted for COVID-19 infection may not have been tested due to low suspicion for CDI and attribution of gastrointestinal symptoms to COVID-19 infection. One obvious difference observed in our study was a rise in CDI during peak periods of the pandemic. This may be attributed to increased antibiotic use, as peak periods of the pandemic over the past year were marked by increased severity of COVID-19 and higher intensive care unit (ICU) occupancy. In addition, either hand washing or use of alcohol-based hand rubs can be used to prevent transmission of COVID-19 whereas strict handwashing would be needed to prevent the spread of CDI. Therefore, it can also be hypothesized that use of alcohol-based hand rubs along with PPE would have effectively curtailed spread of COVID-19, whereas this may not have prevented CDI transmission.

Similarly, a cohort study performed in New York compared CDI rates prior to and during the pandemic. Overall CDI rate was not significantly different between both time periods. Co-infected patients did have an increased length of stay. This study also noted decreased C. diff testing rate during the COVID-19 pandemic. This decrease in testing was likely related to attribution of gastrointestinal symptoms in these patients to COVID-19 infection [9]. A study from Spain also reported similar results. Of the 2300 patients admitted with COVID-19, the incidence of nosocomial CDI was 2.68 per 10,000 patient days which was significantly lower than a rate of 8.54 per 10,000 patient days prior to the pandemic. The reduction in the rate of CDI was highly significant in this study and was likely a result of strict infection control measures [10]. Contrarily, a case series published by Sandhu et al. showed a rise in CDI rate during the pandemic. However, about a third of the patients included in this study had a prior history of CDI and nearly all patients had multiple comorbidities, advanced age, and pre-admission antibiotic exposure [11].

There are several limitations to our study. Overall, we may have not identified all cases of CDI. Patients may have been discharged home with gastrointestinal symptoms that may have been attributed to COVID-19 infection. It is certainly possible that community-acquired CDI may have been mislabeled as nosocomial CDI in our study. However, we do not expect this to impact the overall objective of our study. The objective of this study was strictly to determine rates of CDI. Our study did not evaluate predictors for CDI as certain patient populations may have been at added risk. We did not differentiate between the first episode and recurrence of CDI. Our study did not focus on outcomes in these patients. However, we plan to analyze this in our subsequent work. One additional area of interest that will be addressed in our future study is the changing C. diff isolate pattern. Over the years, NAP1 strains account for over 20% of CDI cases and this proportion does appear to be gradually rising. This may be of further interest in the future as it is likely to impact outcomes in patients with CDI.

Overall, there is limited data on outcomes in patients with COVID-19 and C. diff co-infection. Unexplained diarrhea in the presence of risk factors such as recent antibiotic use should prompt testing for C. diff. Although we can safely conclude that antibiotic use is a modifiable risk factor for CDI, indicated antibiotics should be used without fear of developing CDI. And at the same time, antibiotics should be used judiciously and for appropriate indications only. The overall prevalence of CDI among hospitalized COVID-19 patients was not significantly higher despite the potential widespread use of antibiotics, likely due to aggressive contact and isolation precautions that were implemented during the pandemic.

## Conclusions

COVID-19 infection is often associated with gastrointestinal symptoms such as diarrhea. Antibiotic use was prevalent at the start of the pandemic, which inherently was a risk factor for CDI. Those experiencing diarrhea while admitted for COVID-19 infection may not have been tested due to low suspicion for CDI and attribution of gastrointestinal symptoms to COVID-19 infection. Based on our study, the prevalence of CDI among hospitalized COVID-19 patients was not significantly higher during the pandemic despite the potential widespread use of antibiotics. This is likely due to aggressive contact and isolation precautions that were implemented during the pandemic.
